# Localized biphasic malignant mesothelioma presenting as a giant pelvic wall mass: a rare case report and literature review

**DOI:** 10.1186/s12880-020-00443-w

**Published:** 2020-05-06

**Authors:** Yunsong Liu, Jingjun Wu, Ying Zhao, Pengxin Zhang, Zhengyu Hua, Wan Dong, Tao Lin, Ailian Liu

**Affiliations:** 1 Department of Radiology, The First Affiliated Hospital of Dalian Medical University, Xigang district, Zhongshan road, No.222, Dalian, China; 2 Department of Pathology, The First Affiliated Hospital of Dalian Medical University, Xigang district, Zhongshan road, No.222, Dalian, China

**Keywords:** Biphasic, Malignant peritoneal mesothelioma, Pelvic, Computed tomography (CT)

## Abstract

**Background:**

Localized biphasic MPeM is rare in clinical practice, we reviewed 8 cases of localized biphasic MPeM (including our present case), and summarized the clinical and imaging features of the disease.

**Case presentation:**

We reported a 79-year-old man with chief complaint of a narrowing in the caliber of the stool for one year. A soft tissue shadow was occasionally found by CT examination in the right pelvic wall, and it was diagnosed as localized biphasic malignant peritoneal mesothelioma (MPeM) by postoperative pathology. Radical excision was performed and no radio-chemotherapy was applied. Nearly six years after surgery, the mass was significantly enlarged, and the neighboring tissues including rectum, prostate, seminal vesicle, and right ischial ramus were all infiltrated. The patient was in the end stage of cancer with poor prognosis.

**Conclusions:**

The localized biphasic MPeM may show following characteristics: (1) with heterogeneous low-density and obscure margin; (2) with low incidence rate of ascites; (3) with few central hemorrhage and necrosis; (4) with few calcified structures; (5) with mild to moderate heterogeneous delayed enhancement on contrast-enhanced CT. The imaging characteristics can provide further information for the diagnosis of localized biphasic MPeM in the future.

## Background

Mesothelioma is defined as the transformation of mesothelial cells from the lining of any human cavity into a tumor. The malignant mesothelioma (MM) is relatively rare in clinical practice, and has a highly invasive form. The most common site of MM was the pleura, and MM arising from the peritoneum of the pelvic wall was rarely reported [1]. The distribution of MM is diffuse or localized in two ways. The former is more common and presents as diffuse nodule or mass, the latter is relatively rare and presents as localized mass, which is usually large in size [2]. Histologic classification of MM includes epithelial, sarcomatoid, and biphasic subtypes according to World Health Organization (WHO) [3]. Simple epithelioid MM is the most common histologic type of the disease. Sarcomatoid and biphasic MM are relatively rare. To the best of our knowledge, only seven similar cases [2, 4–9] of localized biphasic malignant peritoneal mesothelioma (MPeM) have been reported.

The present study reported a localized epithelial and sarcomatoid mixed mesothelioma derived from pelvic wall, of which diagnostic and therapeutic experience remain limited. We reviewed the patient’s clinical, imaging, pathological, therapeutic and prognostic information in order to provide more clues for this disease.

## Case presentation

A 79-year-old man came to our gastrointestinal outpatient with complaint of a narrowing in the caliber of the stool without obvious cause for about 1 year. Otherwise, he had no history of asbestos exposure, hematochezia, diarrhea, constipation, abdominal pain and distention. He had a history of prostatectomy due to benign prostatic hyperplasia and he denied recent weight loss. Digital examination of rectum showed the lower boundary of a mass in the rectum was 2 cm from the anal margin, and the upper boundary could not be palpated. Therefore, the patient underwent colonoscopy endoscopic electrocoagulation resection.

**Fig. 1:**
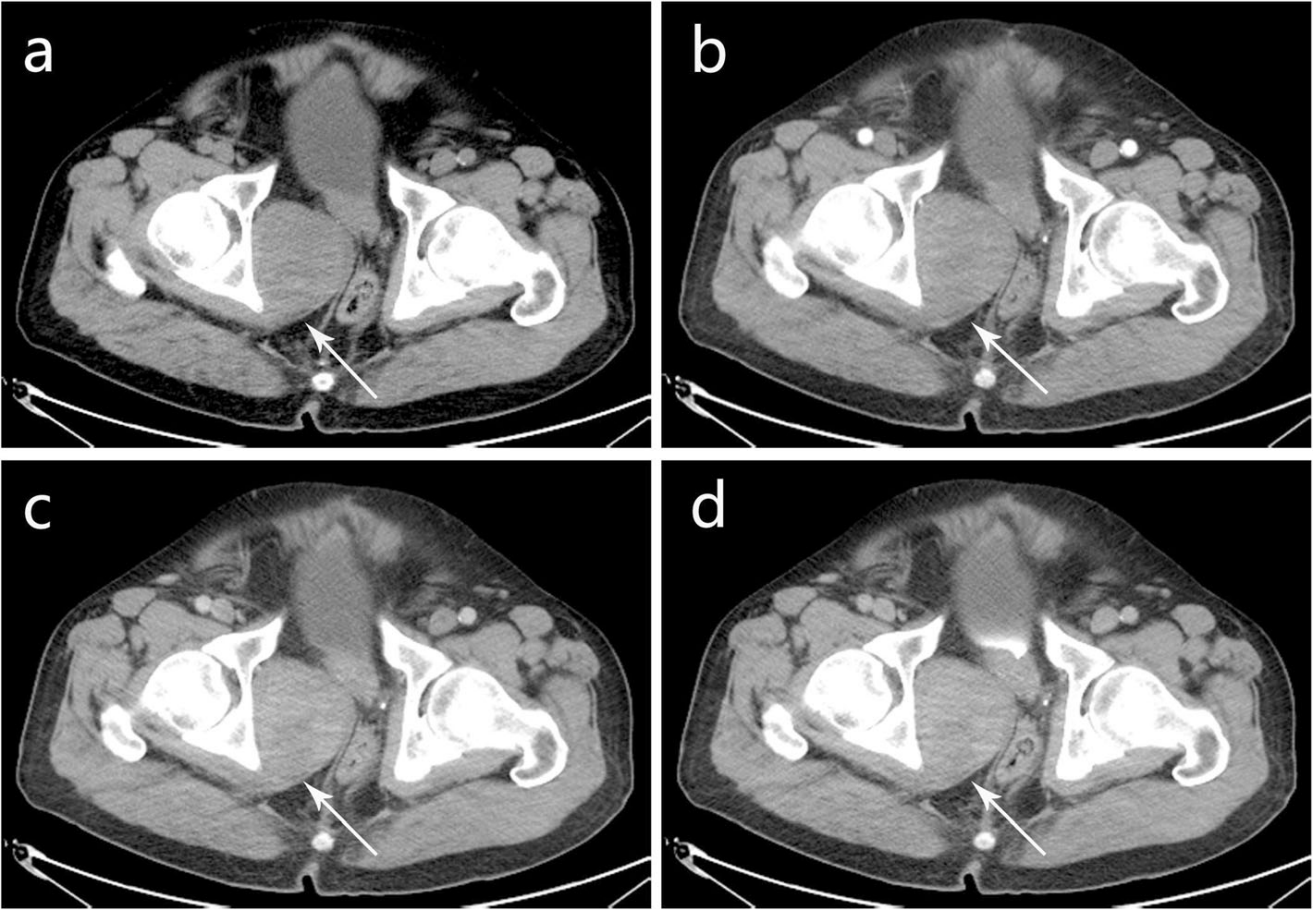
(**a**) Abdominal plain CT scan revealed an slightly heterogeneous soft tissue mass (white arrow) with well-defined boundary. The mass showed mild heterogeneous enhancement in (**b**) arterial, (**c**) venous, (**d**) and delayed phase

A soft tissue shadow was occasionally found by pelvis CT examination in the right pelvic wall. The mass had well-defined boundary and was oval shape with size of 7.3 × 5.3 cm. No calcification was found within the mass. On unenhanced CT images, the mass was heterogeneous with suspected necrotic area (Fig. 1a). In arterial, venous and delayed phases, CT values were 32–61 Hu, 65–90 Hu, and 46–77 Hu, respectively. The lesion showed mild heterogeneous on delayed enhancement CT images (Fig. 1b-d). Otherwise, the rectum and prostate were pressed, and no sign of destruction was observed in the adjacent bone. The CT examination suggested that the mass may originate from striated muscles with malignant transformation, and it may belong to neurogenic benign tumor. The source of blood supply to the mass was identified by pelvic computed tomography angiography (CTA), which showed the mass was mainly supplied by the right internal iliac artery (Fig. 4b). Unfortunately, the local dissection of the right common iliac artery was observed by CTA, and delayed the treatment of pelvic mass. Then, the right common iliac artery dissection was treated in other hospital more than a month later.

Six month after the discovery of the pelvic mass, the mass was slightly larger observed by CT examination (Fig. 4a). The patient underwent radical excision of pelvic mass. Intraoperatively, a solid mass with complete capsule was disclosed at the right obturator site of the pelvic wall. The lesion was 8 × 6 cm in size, with nodular surface and well-defined boundary. The surgeons removed the mass completely. The patient did not receive radio-chemotherapy and was in good condition after surgery.

**Fig. 2:**
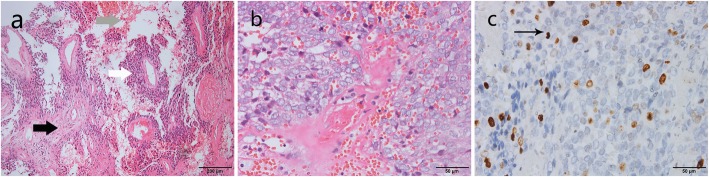
Peritoneal biphasic malignant mesothelioma subtypes was shown on hematoxylin and eosin-stained sections. **a** The biphasic pattern is characterized by epithelioid cells with papillo-tubular structure (white arrow) and sarcomatoid components (black arrow). Bleeding was observed in some areas (gray arrow) (original magnification × 100). **b** Sarcomatoid components have hypercellular short shuttle-like or round cell, which arranged disorderly (original magnification × 400) **c** Immunohistochemistry showed the positive rate of Ki-67 was less than 10% (original magnification × 400), which suggests that tumor cell proliferation is relatively inactive

**Fig. 3:**
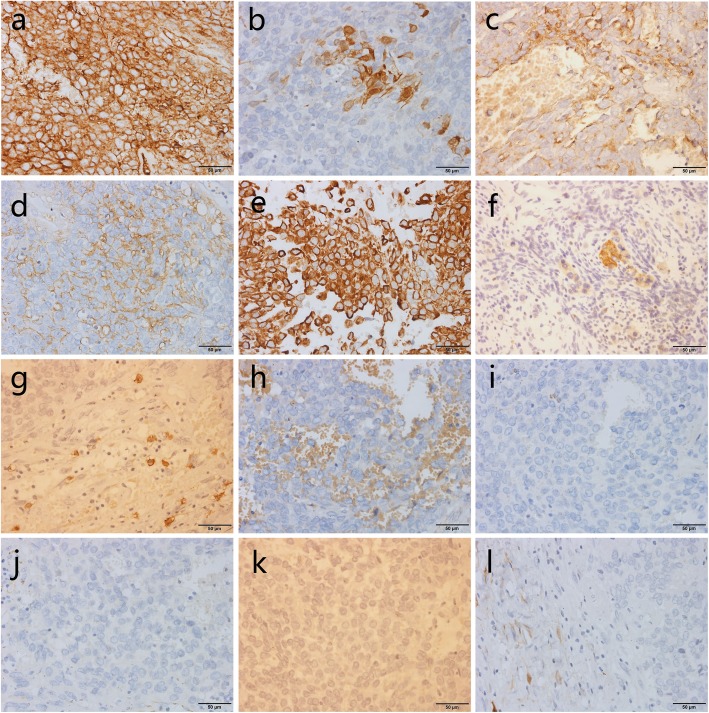
Immunohistochemistry (original magnification × 400) showed that tumor cells were positive for (**a**) CD34, (b) calretinin, (**c**) EMA, (**d**) MC, (**e**) Vimentin, and negative for (**f**) CD99, (**g**) CD117, (**h**) CK5/6, (**i**) CK7, (**j**) CK20, (**k**) HMB45, (**l**) S-100

The postoperative pathological examination showed that the mass was biphasic differentiated to both epithelium and mesenchyma (Fig. 2a-b). Immunohistochemistry is also important for the diagnosis of the mass. The ki-67 was less than 10%, which suggested that tumor cell proliferation is relatively inactive (Fig. 2c). Mesothelioma cells were positive for CD34, calretinin, EMA, MC and Vimentin, and negative for CD99, CD117, CK5/6, CK7, CK20, HMB45 and S-100 (Fig. 3a-l). In summary, the tumor was considered as biphasic malignancy mesothelioma.

**Fig. 4:**
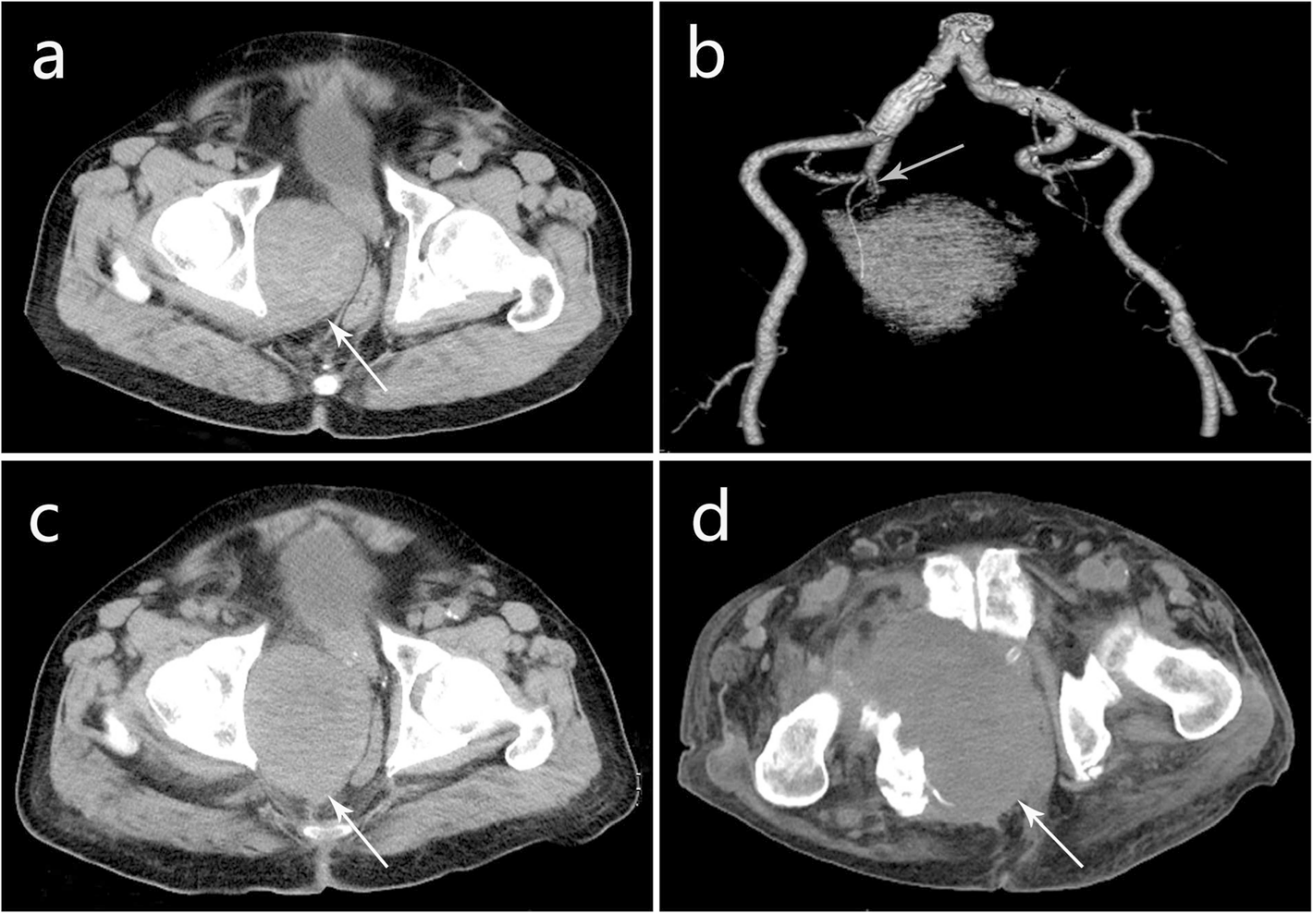
The group of pictures showed a preoperative mass and lesion recurrence (white arrow). **a** The mass (preoperative) was slightly enlarged compared to the size examined by first-time CT imaging. **b** CTA showed the right internal iliac artery was the main blood supply to the mass (gray arrow). **c** Lesion recurrence was first detected four years after surgery. **d** Lesion was significantly enlarged nearly six years after surgery

One and a half years after surgery, the patient underwent pelvic CT reexamination and no sign of tumor recurrence was found. Four years after surgery, the patient attended a local hospital due to progressive dysuria and lower abdominal pain. Then pelvic CT showed a soft tissue mass in the preexisting position and the mass was measured as 7.89 × 10.41 cm with oval shape and well-defined boundary (Fig. 4c). We still saw that the mass had heterogeneous density in unenhanced CT scan. In the arterial, venous and delayed phases, CT values were 36 Hu, 58 Hu, and 60 Hu, respectively. The mass showed mild to moderate heterogeneous delayed enhancement. The right ischium was destroyed. Neighboring tissues including rectum, prostate and seminal vesicle were all infiltrated and pressed by the mass. Then, the patient underwent three times chemoembolization successively, and he was significantly relieved of symptoms after treatment. Nearly 6 years after surgery, the patient came to our hospital for further palliative treatment of the tumor. CT reexamination revealed the mass had unclear boundary and significantly enlarged. The bone destruction of right ischial ramus was observed. Scattered and irregularly distributed patchy calcifications were observed in the mass, which may be a hypertrophic response caused by bone destruction. The walls of bladder, descending colon, and sigmoid colon were thickened due to tumor invasion (Fig. 4d). In addition, there was a peritoneal effusion. Finally, the patient was in the last stage with cachexia, and his prognosis was poor.

## Discussion and conclusion

Our present case was a localized biphasic MM originated from the peritoneum of the pelvic wall. We made a detailed analysis about relevant publications, and our present case was also included (Table 1). The cases consisted of five males and three females (1.67: 1). In industrialized countries, the prevalence rates of MPeM ranged from 0.5 to 3 cases per million in men and from 0.2 to 2 cases per million in women [10]. So men may have a higher incidence than women. In Kawai et al. ‘s study [11], the ratio of men to women was 4.5 to 1, which is similar to our study. Patients were aged from 41 to 79 years, and the median age was 69 years old. The tumors appear to affect mainly the older population. Localized tumor in the liver was observed in 5/8 cases (62%), 2/8 (25%) in the abdominal wall, and 1/8 (13%) in the transverse colon. At present, the epidemiology of malignant peritoneal mesothelioma is more ambiguous than that of pleural mesothelioma. The epidemiology of MPeM varies with various factors. Asbestos exposure is the main cause of MPeM. But patients with peritoneal mesothelioma are less likely to have a well-defined history of asbestos exposure than patients with pleural mesothelioma [12]. Only 2 patients (25%) had a history of asbestos exposure. Typical initial symptoms of MPeM were abdominal pain, abdominal distention, or weight loss [13]. Initial symptom of 3 patients (38%) was abdominal pain. Four patients (50%) had no obvious symptoms. One (12%) patient noticed enlarging lump in right abdominal wall. Most patients showed normal results about biochemistry examinations. However, 4 patients (50%) had anemia on hematologic examinations, which may be due to bleeding from lumps. Tumor markers such as carcino-embryonic antigen (CEA), alpha fetoprotein (AFP), CA12–5, CA19–9 were normal in the 8 cases. Most of the cases reported as the localized tumors were with a median size of 11.2 cm (range 4–24 cm). One case had a very large mass,which occupied the right abdominal cavity and bilateral pelvic cavity.

**Table 1:** Literature review and clinical data analysis

Author/year	Age	Sex	Asbestos Exposure	Location	Size (cm)	Anemia	Initial symptom
Sasaki et al [4]/2009	66	Male	Yes	Liver	4	No	No obvious symptoms
Shao et al [2]/2011	77	Female	No	Right abdominal wall	Very large	No	Notice enlarging lump
Kohno et al [5]/2012	69	Male	Yes	Left abdominal wall	10.7	No	No obvious symptoms
Takehara et al [6]/2014	72	Male	No	Transverse colon	10	Yes	Abdominal pain
Serter et al [7]/2015	66	Male	No	Liver	20	Yes	Abdominal pain
Ali et al [8]/2016	41	Female	No	Liver	24	Yes	No obvious symptoms
Dalal et al [9]/2018	69	Female	No	Liver	9	Yes	Abdominal pain
Present case	79	Male	No	Liver	8	No	No obvious symptoms
Author/year	Tumor marker		Treatment		Follow-up		
Sasaki et al [4]/2009	Normal		Radical excision		No recurrence or metastasis 6 months after surgery		
Shao et al [2]/2011	Normal		Symptomatic treatment		Died 6 months after discovery		
Kohno et al [5]/2012	Normal		Radical excision		No recurrence more than 7 months after operation		
Takehara et al [6]/2014	Normal		Radical excision		Died 6 months after operation		
Serter et al [7]/2015	Normal		Radical excision		Unknown		
Ali et al [8]/2016	Normal		Radical excision		Unknown		
Dalal et al [9]/2018	Normal		Radical excision and adjuvant chemotherapy		Recurrence and progression during follow-up		
Present case	Normal		Radical excision		Recurrence 4 years after surgery		

Next, we reviewed the immunohistochemical data of all present cases. In Table 2, mesothelioma cells were positive for calretinin in 8/8 (100%) cases, vimentin in 7/7 (100%), CK5/6 in 5/6 (83%), WT-1 in 3/5 (60%) and negative for CK20 in 3/3 (100%), HMB-45 in 3/3 (100%), S-100 in 4/4 (100%). So we found that the most dominant group of positive markers were calretinin, vimentin, CK5/6. Meanwhile, the most significant group of negative markers were CK20, HMB-45, S-100.

**Table 2:** Literature review and data analysis about radiological data

Author/year	Central hemorrhage and necrosis	Calcification	Heterogeneous low-density	Enhanced mode	poorly-defined margins	Ascite
Sasaki et al [4]/2009	Yes	No	Yes	peripheral staining	No	No
Shao et al [2]/2011	Yes	No	Yes	mild to moderate heterogeneous delayed enhancement	Yes	Yes
Kohno et al [5]/2012	Yes	No	Yes	peripheral staining	Yes	No
Takehara et al [6]/2014	No	No	No	peripheral staining	Yes	No
Serter et al [7]/2015	Yes	No	Yes	peripheral staining	Yes	No
Ali et al [8]/2016	Yes	Yes	Yes	Unknown	No	No
Dalal et al [9]/2018	Yes	No	Yes	Unknown	Yes	No
Present case	No	No	Yes	mild to moderate heterogeneous delayed enhancement	No	Yes

At present, immunohistochemistry has been commonly used to diagnose malignant mesothelioma. However, CT, a commonly used diagnostic method for abdominal lesions, shows no specific manifestation in the diagnosis of MPeM. In the study of 244 MPeM cases, Tandon et al. found that the most sensitive immunohistochemical markers were calretinin (100%), WT1 (94%), and CK5/6 (89%) [14], which was similar to our study. Saito et al. believed that calretinin, CK 5/6, mesothelin, vimentin, epithelial membrane, and WT-1 were specific markers of tumor mesothelial origin [15]. Firstly, MPeM should be distinguished from similar benign lesions, such as reactive mesothelioma and mesenteritis. Kawai et al. found that EMA, P53, desmin and p-glycoproteins were 100% expressed in malignant pleural mesothelioma, but no positive marker was found in the cases of reactive mesothelioma [11]. One of the most effective methods to distinguish MPeM and reactive mesothelial hyperplasia was fluorescence in situ hybridization (FISH), which could be used to analyze the homozygous deletion at site 9p21, which was positive in 67% of pleural mesothelioma, but the positive rate of peritoneal mesothelioma was low, only 25% [16]. Therefore, this method was not applied in our case. Liang et al. [17] found that the pattern of peritoneal thickening and contrast-enhanced imaging were effective markers for differentiating MPeM and peritoneal carcinomatosis, but their case was DMPeM (Diffuse MPeM), so it was of little help in differentiating this case. Liang et al. also found that on CT images, the mesenteric lipomatosis showed soft tissue nodules, perivascular fatty halo and nodules, which may be helpful to distinguish MPeM from mesenteric lipomatosis. Malignant diseases include metastatic peritoneal adenocarcinoma and rhabdomyosarcoma were similar to MPeM. Peritoneal carcinoma was a metastatic feature of many organ malignancies, especially of the gastrointestinal tract and ovary, and must be considered as a first possibility even in the absence of a clear primary focus [17]. The most common malignancy reported by Walkey et al. was ovarian cancer [18]. Metastatic peritoneal adenocarcinoma is histologically difficult to distinguish from MPeM. Kawai et al. found that the best negative mesothelioma markers to distinguish epithelioid mesothelioma from serous carcinoma were be-ep4 and moc-31, and the best positive mesothelioma markers were d2–40 and calretinin [11]. The primary lesion of peritoneal adenocarcinoma found on CT is also a strong evidence for the diagnosis of metastatic peritoneal adenocarcinoma. Arora et al. found that a specific myogen, a muscle-derived marker, could rule out rhabdomyosarcoma if it was negative [19]. However, biphasic MPeM contained sarcoma components, so it was difficult to exclude rhabdomyosarcoma by relying on it alone. It required a combination of specific markers of epithelial and mesenchymal origin for a comprehensive analysis. In a word, immunohistochemical diagnosis of MPeM is progressing well, but there are still many problems. Due to the small number of cases, few specific imaging findings were found.

Therefore, we collected radiological data from all the biphasic MPeM cases of restricted growth patterns available at present. Radiological studies play an important role in the diagnosis, staging and prognosis of biphasic MPeM. Among them, the contrast-enhanced CT is the major imaging modality for MM [20]. CT has an advantage in distinguishing between biphasic MPeM and its surrounding tissues in order to observe whether there is pathological infiltration. In addition, CT has the ability to display images over a wide range and to clearly show lumps in different areas, which is helpful in finding the origin of biphasic MPeM. Because biphasic MPeM is extremely rare, there is currently few imaging description of localized biphasic MPeM. A review of eight cases was summed up about radiological data, and our present case was also included in Table 2. The masses presented as heterogeneous low-density lesion on non-contrast CT scan in 7/8 cases (88%). One case presented as homogeneous low density tumor. On dynamic enhanced CT scan, the lesions presented as peripheral staining in 4/6 (66.7%) and mild to moderate heterogeneous delayed enhancement in 2/6 (33.3%). Tumors with obscure margin were seen in 5/8 (63%) cases. Only one case had few small calcifications, and 75% cases (6/8) developed hemorrhage and necrosis in the center. In the present case, CT showed no obvious hemorrhage and necrosis in the center of the mass as shown in Fig. 1a, showing only a slightly heterogeneous density within the lesion. Although the histopathological image showed a small amount of extravasation of red blood cells in Fig. 2a, this didn’t definitively demonstrate significant bleeding in the central area of the lesion. Only two (25%) cases had ascites. We found no other significant imaging features.

Currently, no standard treatment of malignant peritoneal mesothelioma had been established, and localized MPeM has been usually treated with radical resection. In Table 1, all cases presented as localized tumor in the peritoneum at initial diagnosis. Radical excision was performed in seven cases, and only one patient undertook symptomatic treatment because the lesion was too large. In addition to radical resection, only one patient underwent postoperative adjuvant chemotherapy. According to the analysis of follow-up data, we perceived that the prognosis was variable: two cases had no recurrence less than a year after surgery, two cases were recurrent postoperatively, and two cases were dead less than a year after surgery. Our patient experienced a period of nearly seven years from biphasic MM discovery to the last time follow-up, which demonstrated a relatively good prognosis. We suspected that the prognosis of localized biphasic MPeM was generally poor, and early treatment was urgently necessary.

At present, the diagnosis of MM is still difficult, and the diagnostic standards are usually pathological examination including immunohistochemistry. Although imaging examination has only made little progress in the diagnosis of MM, it can still show the spatial or temporal features of mass with a non-invasive way compared with pathology. The diagnostic efficacy of radiological examination for MM is improving by reviewing more cases of MM. Histology and immunohistochemistry also have limitations in the classification of subtypes of MM. The classification of subtypes of MM by imaging has been explored recently [21–23]. For example, Escalon ea. al [21]. found the calcified pleural plaques and local invasion were more common in non-epithelioid subtypes of malignant pleural mesothelioma. Similar studies in the abdomen and pelvic need be further carried out in the future.

In summary, the present case and literature review suggest that the localized biphasic MPeM may show following characteristics: (1) with heterogeneous low-density and obscure margin; (2) with low incidence rate of ascites; (3) with few central hemorrhage and necrosis; (4) with few calcified structures; (5) with mild to moderate heterogeneous delayed enhancement on contrast-enhanced CT. We hope that our report on localized biphasic MPeM will provide further information for the diagnosis, classification and treatment of the disease in the future.

## Data Availability

All data generated or analyzed during this study are included in this published article.

## Abbreviations

AFP: Alpha fetoprotein
CEA: Carcino-embryonic antigen
CTA: Computed tomography angiography
MPeM: Malignant peritoneal mesothelioma
MM: Malignant mesothelioma
WHO: World health organization
